# Treadmill Exercise Reverses Depression Model-Induced Alteration of Dendritic Spines in the Brain Areas of Mood Circuit

**DOI:** 10.3389/fnbeh.2019.00093

**Published:** 2019-05-03

**Authors:** Pu-Chao Zhuang, Zhi-Nei Tan, Zi-Yan Jia, Biju Wang, James J. Grady, Xin-Ming Ma

**Affiliations:** ^1^College of Life Sciences, Shaanxi Normal University, Xi’an, China; ^2^Department of Statistics, University of Connecticut, Storrs, CT, United States; ^3^Connecticut Convergence Institute, University of Connecticut Health, Farmington, CT, United States; ^4^Department of Community Medicine, University of Connecticut Health, Farmington, CT, United States; ^5^Department of Neuroscience, University of Connecticut Health, Farmington, CT, United States

**Keywords:** depression-like behaviors, chronic unpredictable mild stress, spine density, hippocampus, medial prefrontal cortex, nucleus accumbens

## Abstract

Depression is one of the most prevalent psychiatric disorders. Exercise has been shown to be effective in the amelioration of depression, but the underlying mechanism remains largely unknown. Alterations in the density and morphology of dendritic spines are associated with psychiatric diseases. Chronic unpredictable mild stress (CUMS) is an established animal model of depression. The aim of this study was to determine whether treadmill exercise reverses CUMS-induced both depression-like behaviors and alterations in spine density and morphology of the principal neurons in the brain areas of the mood circuits including the hippocampus, medial prefrontal cortex (mPFC), nucleus accumbens (NAc) and basolateral amygdala (BLA). Male rats were randomly divided into four groups: control, CUMS, exercise, and CUMS+exercise. CUMS-induced depression-like behaviors were evaluated by the sucrose preference test (SPT). Golgi staining was used to visualize dendritic spines. Our results showed that CUMS-induced depression-like behaviors characterized by a decrease in sucrose consumption were accompanied by a decrease in spine density and a change in spine morphology in the pyramidal neurons of both the hippocampal CA3 area and the mPFC, and an increase in spine density and an alteration in spine shape in both the NAc medium spiny neurons (MSNs) and the BLA neurons; exercise reversed both CUMS-induced depression-like behaviors and alterations in dendritic spines. This study provides important information for understanding the mechanism through which exercise ameliorates CUMS-induced depression-like behaviors.

## Introduction

Depression, a serious mood disorder, is characterized by sadness, anhedonia, loss of interest, disturbed sleep, feelings of hopelessness and fatigue. It is now the leading cause of ill health and disability worldwide and is a major contributor to the overall global burden of disease according to the WHO^1^. Although depression has been studied for decades, the underlying mechanisms are still largely unknown. Currently available antidepressant drugs show low efficacy and slowness of onset, with serious side effects; many patients are resistant to current treatments (Akil et al., 2018; Wang et al., 2018). Therefore, a deeper understanding of the underlying mechanisms might provide further insight to drive the development of more effective therapeutic approaches.

Depression in humans and depression-like behaviors in animal models are associated with structural and functional abnormalities in the same brain areas as the mood circuits including hippocampus, prefrontal cortex (PFC), nucleus accumbens (NAc) and amygdala (Drevets et al., 2008; Russo and Nestler, 2013; Qiao et al., 2016). Depressed patients have a smaller hippocampus, PFC and NAc compared with healthy controls (Drevets et al., 1997; Colla et al., 2007; Pizzagalli et al., 2009). There are conflicting reports on amygdala volume in depressed patients (Rubinow et al., 2016; Qiao et al., 2016). Importantly, hippocampal atrophy in depressed patients is associated with greater depression severity (Taylor et al., 2014). The medial PFC (mPFC), the functional center of the PFC, is divided into three regions including the anterior cingulate cortex (ACC), prelimbic cortex (PL) and infralimbic cortex (IL). Abnormal structure and function in the mPFC are involved in depression (Drevets et al., 2008). The smaller PFC is accompanied by a decrease in the expression of synaptic proteins and loss of spine synapse number in the PFC in MDD subjects compared with controls (Kang et al., 2012). The NAc, the center of the brain reward circuit, plays an important role in modulating mood and emotion and is associated with depression (Russo and Nestler, 2013). The structure of basolateral amygdala (BLA), a region of amygdala involved in emotion, mood, and motivation, is altered in depression (Rubinow et al., 2016).

Dendritic spines, small protrusions on the surface of dendrites, are a highly dynamic and important structure for neuronal connections, information storage and processing within the brain circuits. Although it remains unknown if there is a direct connection between altered dendritic spines in human brain and depression, preclinical studies suggest that alterations of spine density and morphology in the principal neurons of brain areas on the mood circuits are associated with depression (Duman and Duman, 2015; Qiao et al., 2016). A deeper understanding of dendritic spine abnormalities in these brain regions using an animal model of depression might greatly enhance our understanding of depression.

Chronic unpredictable mild stress (CUMS), an established animal model of depression (Willner, 2017), induces depression-like behaviors accompanied by a decrease in spine density in the principal neurons of the hippocampus and mPFC in rodents (Li et al., 2011; Qiao et al., 2014). However, it remains unclear whether CUMS alters spine density in both the NAc medium spiny neurons (MSNs) and the neurons of the amygdala.

Compared with traditional antidepressants, exercise is more economic and does not have the unwanted side effects. Clinical studies have demonstrated that exercise is effective to ameliorate depression (Belvederi Murri et al., 2019). However, some studies show that exercise fails to improve cognition symptoms in major depressive disorder (Brondino et al., 2017; Sun et al., 2018). Recent preclinical studies show that exercise alleviates CUMS-induced depressive-like behaviors in rodents (Chen et al., 2017; Liu et al., 2018), but the underlying mechanism is not clear. The aim of this study was to determine whether exercise reverses both CUMS-induced alterations in dendritic spines in brain areas including the hippocampus, mPFC, NAc and BLA and the accompanying depression-like behaviors.

## Materials and Methods

### Animals

The animal protocol of the study was approved by the Animal Care and Use Committee of Shaanxi Normal University. The investigation was conducted in accordance with the ethical principles of animal use and care. Adult male Sprague-Dawley rats, weighing 225–250 g (8 weeks) at the beginning of the experiment, were purchased from the Xian Jiaotong University. The rats were kept under a 12 h light/12 h dark cycle at a suitable temperature (23 ± 3°C) and humidity (55 ± 10%) with free access to food and water. Animals were adapted to the laboratory conditions for 6 days before starting the experiment. Animals were randomly divided into four groups: control, exercise (EX), CUMS and CUMS+EX (*n* = 8–9).

### CUMS Procedure

CUMS procedure was performed as described (Li et al., 2011; Qiao et al., 2014) with slight modification. Each rat was kept in one cage and received 3 weeks of stress. In the first week, stressors were applied once a day; in the second week, two stressors were applied every other day; in the third week, each animal received two random stressors per day. The stressors were: cold water swimming (5 min at 4°C), hot water swimming (5 min at 45°C), tail nip for 1 min, cage shaking for 30 min, lights off for 3 h, food and water deprivation overnight, wet bedding overnight, lights on overnight, tilted cage (45°) overnight, crowded group housing overnight, and strobe lights overnight. The same stressor was not applied in two consecutive days. Control animals did not receive exercise or CUMS, and were daily handled for 21 days.

### Exercise Protocol

**Figure 1:**
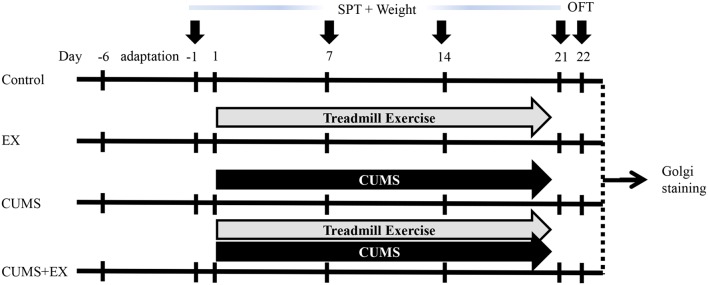
Experimental design. Body weight and sucrose preference were measured on the day −1, 7, 14 and 21. Open field test (OFT) was performed on day 22. Brains were collected for Golgi staining on day 23. CUMS, chronic unpredictable mild stress; SPT, sucrose preference test; EX, treadmill exercise.

Animals in the EX and CUMS+EX groups received one period of six-lane treadmill exercise (Anhui Zhenghua Biological Instrument Equipment Co, Limited) per day from Monday to Friday for 3 weeks as described (Kiuchi et al., 2012; Wen et al., 2014) with some modifications (Figure 1). All rats did not receive exercise on Saturday and Sunday. In the first week, rats were trained on the treadmill for 20 min at the speed of 10 m/min (0°), with VO_2_ max approximately 50%–55%. In the second and third weeks, rats were trained for 30 min at 15 m/min (0°), with VO_2_ max (maximal oxygen uptake) approximately 60%–65%. The exercise procedure was applied at 9:00 am. In the CUMS+EX group, exercise and stress were performed separately and the resting interval between the two procedures was at least 1 h.

### Body Weight and Behavioral Tests

Body weight assessment and the sucrose preference test (SPT) were performed as described (Qiao et al., 2014; Liu et al., 2018; Figure 1). The SPT was used to measure sucrose consumption (anhedonia, a typical depression-like behavior) on the day before the onset of CUMS, and on days 7, 14 and 21 after the onset of CUMS. Body weight was accessed as the percentage of body weight gain on days 7, 14 and 21 over the initial body weight before the onset of exercise and CUMS. The first SPT was performed on the day before starting CUMS. For training purposes, at 16:00 2 days before the onset of CUMS, each rat was kept alone in a small cage and given two bottles of 1% sucrose at 20:00 for 12 h until 8:00 am the next day. Then each rat was given a bottle of water and a bottle of 1% sucrose solution for 4 h. After training, rats were deprived of water for 6 h from 12:00 to 18:00. For measuring the SPT, each rat was given one bottle of water and one bottle of 1% sucrose for 4 h from 18:00 to 22:00, and the position of the two bottles was exchanged at 20:00 to avoid position preference. The weight of water and 1% sucrose was measured before and after the SPT, respectively, and calculations were made as the sucrose preference (%) = sucrose consumption/(sucrose consumption + water consumption). The other three SPTs on days 7, 14 and 21 were performed without training. Open field test (OFT) was performed on day 22 to measure activity, the exploratory and anxiety-related behaviors. The moving trace was recorded by a camera operated by a computer. Rearing and grooming times were recorded by a researcher blind to the experiment.

### Golgi Staining and Dendritic Spine Density Analysis

One day after behavioral tests, rats were fixed for Golgi staining (*n* = 5–6) as described (Qiao et al., 2014). Briefly, rats were fixed by perfusion with 4% paraformaldehyde, whole brains were immersed in Golgi-Cox solution for 14 days in the dark and then were transferred to 30% sucrose solution until they sank to the bottom of the container. Vibratome slices of 150 μm in thickness were sequentially processed through dH_2_O (1 min), 28% ammonium hydroxide (40 min in the dark), dH_2_O (1 min), acidic hardening solution (40 min, in the dark) and dH_2_O (2 × 1 min). Finally, slices were dehydrated and cleared through ascending concentrations of alcohol and xylene before being mounted on slides.

Z-stack images were collected using a light microscope (Axio Observer.Z1 with ApoTome2, Zeiss, Germany), and the optimal Z-step was recommended by the software (ZEN 2.3, Zeiss, Germany). One dendrite per neuron and at least eight neurons per brain area were randomly imaged. In the pyramidal neurons of the CA3, ACC, PL and IL, a second-order segment in the apical dendrite 100 μm away from the soma was imaged (Radley et al., 2006; Qiao et al., 2014). In the NAc core and shell, a segment in the dendrite of the MSN 50 μm away from the soma was imaged (Christoffel et al., 2011). In the BLA neurons, a second-order segment of the dendrite 50 μm away from soma was imaged (Mitra et al., 2005). All images were taken with identical settings under the same conditions. Z-stack images were collapsed to a single image using NIH ImageJ. The image was then opened in MetaMorph (Molecular Devices, San Jose, CA, USA) and an inclusive threshold was adjusted to outline spines. Spine quantifications were performed using MetaMorph (Ma et al., 2008). A spine is defined as a protrusion whose length is no longer than three micrometers. Spine density was quantified after images were calibrated and thresholds were set to ensure that all interesting structures were included in the analysis. To count spine types, spines were classified by the following criteria as described (Qiao et al., 2016): spines are defined as thin if the spine length is greater than its neck diameter and the diameters of the head and neck are equal. Spines are considered mushroom if the diameter of the spine head is greater than the diameter of the neck. Spines are considered stubby if the length and width of a spine are equal. The images were collected by a researcher blind to the experiment.

### Statistical Analysis

The measures of body weight over time were analyzed using a repeated measures general linear mixed model (GLMM) with factors of exercise (yes vs. no), CUMS (yes vs. no) and time (days 7, 14, and 21) using the MIXED procedure in SAS^®^ (SAS, 2004) with the SLICE option to assess pairwise tests using Tukey’s *post hoc* procedure for multiple comparisons. For sucrose preference, OFT and spine density, the analysis was performed with two-way analysis of variance (ANOVA) using the GLM procedure in SAS^®^ with factors of exercise (yes vs. no) and CUMS (yes vs. no) and applying the SLICE option to assess pairwise (Tukey’s) tests when the interaction was significant. Data were represented as the mean ± SEM. A two-sided alpha level of 0.05 was used to assess statistical significance. We analyzed the data using SAS/STAT^®^ 9.4 software.

## Results

### Effect of Treadmill Exercise and CUMS on Body Weight Gain

**Figure 2:**
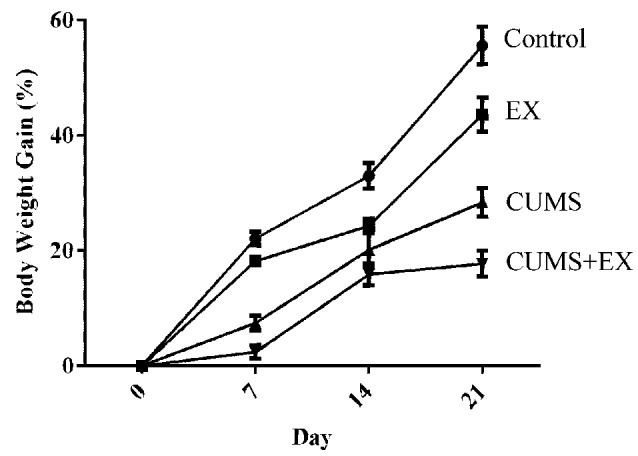
The effect of CUMS and exercise on rat body weight gain. Two-way analysis of variance (ANOVA) analysis showed that 21-day CUMS and exercise had significant effects on body weight gain for exercise (*P* = 0.0001), CUMS (*P* < 0.0001), time (*P* < 0.0001), (CUMS × time; *P* < 0.0001), and (exercise × time; *P* = 0.007). CUMS and exercise were significantly different from each other at each time point (all Tukey adjusted *P* < 0.05). After 21-day CUMS and exercise, both CUMS and treadmill exercise decreased body weight gain in control animals, respectively (*P* < 0.05), but the extent of decrease induced by CUMS was larger than those induced by exercise (*P* < 0.05). Data represent mean ± SEM. Control: control rats received brief handle for 21 days. EX, treadmill exercise group. CUMS, chronic unpredictable mild stress group. CUMS+EX: CUMS-exposed rats received exercise (*n* = 8–9 rats/group).

The total body weight gains were used to evaluate the effect of treadmill exercise and CUMS on body weight (Figure 2). We fitted a repeated measures GLMM with factors of exercise (yes vs. no), CUMS (yes vs. no) and time (days 7, 14, and 21) to the body weight data. Interaction terms of (CUMS × time) and (exercise × time) were added to the model to assess the extent to which the effects of CUMS and exercise affected body weight similarly or differently across time. The model had significant effects for exercise (*P* = 0.0001), CUMS (*P* < 0.0001), time (*P* < 0.0001), (CUMS × time; *P* < 0.0001), and (exercise × time; *P* = 0.007). The next step involved comparing CUMS and exercise, separately, at each time point. CUMS and exercise were significantly different from each other at each time point (all Tukey adjusted *P* < 0.05). The results showed that both the CUMS and treadmill exercise had a significant effect on body weight.

### Effect of Treadmill Exercise and CUMS on Sucrose Preference

**Figure 3:**
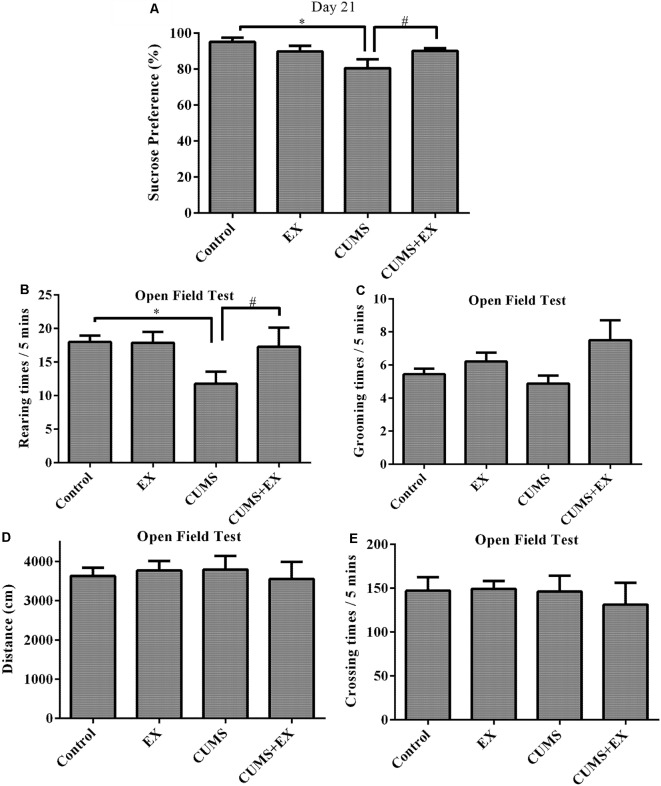
Sucrose preference test (SPT; **A**). Treadmill exercise reversed the CUMS-induced decrease in sucrose consumption on day 21. OFT **(B–E)**. **(B)** Rearing times. Treadmill exercise reversed CUMS-induced decrease in rearing times. **(C)** Grooming times. **(D)** Locomotor activity. CUMS did not affect locomotor activity. **(E)** Crossing times. Data represent mean ± SEM. *P < 0.05 CUMS vs. control, ^#^*P* < 0.05 CUMS vs. CUMS+EX. EX: treadmill exercise. CUMS: chronic unpredictable mild stress. CUMS+EX: CUMS+treadmill exercise (*n* = 8–9 rats/group).

The SPT is used to evaluate anhedonia (a decrease in sucrose consumption), a typical depression-like behavior in rodents (Figure 3A; Bessa et al., 2013). To determine the effects of CUMS/exercise and interactions between CUMS and exercise on sucrose consumption, we performed two-way ANOVA. On day 21 after CUMS and exercise, CUMS and exercise had a significant effect on sucrose consumption (*F*_(3,29)_ = 7.64, *P* = 0.007). CUMS is a significant factor (*P* = 0.003) and so is the interaction term EX*CUMS (*P* = 0.003). In order to analyze how this interaction between exercise and CUMS is significant, we then fixed one level of a factor and analyze if the remaining factor is significant. Our analysis showed that without exercise, CUMS caused a significant decrease in sucrose consumption compared with a control group (*P* < 0.001); with exercise, CUMS did not alter sucrose consumption compared with a control group (*P* < 0.001). By contrast, without CUMS, exercise alone did not alter sucrose consumption (*P* = 0.21); with CUMS, exercise caused a significant increase in sucrose consumption compared with CUMS group (*P* = 0.002). These results showed that exercise reversed the CUMS-induced decrease in sucrose consumption and that the CUMS protocol was sufficient to induce depression-like behavior. Exercise prevented animals from developing depression-like behavior.

### Effects of Treadmill Exercise and CUMS on Behaviors in the Open Field Test (OFT)

The OFT was used to evaluate activity, anxiety-related behavior and exploratory behavior in rodents by recording moving distance, rearing times, grooming times (Figure 3).

Rearing times: exercise and CUMS had the significant effect on rearing times (*F*_(3,18)_ = 3.8, *P* = 0.049). Without exercise, CUMS caused a significant increase in rearing times compared with a control group (*P* = 0.02); with exercise, CUMS did not alter rearing times (*P* = 0.82). Similarly, without CUMS, exercise did not have a significant effect on rearing times (*P* = 0.96); with CUMS, exercise caused a significant increase in rearing times compared with CUMS group (*P* = 0.048). These results showed that exercise reversed the CUMS-induced decrease in rearing times (Figure 3B).

Exercise and CUMS did not significantly affect grooming times (*F*_(3,29)_ = 2.20, *P* = 0.11; Figure 3C), moving distance (*F*_(3,29)_ = 0.13, *p* = 0.94; Figure 3D) or crossing times (*F*_(3,29)_ = 0.22, *p* = 0.88; Figure 3E).

### Dendritic Spines in the Hippocampal CA3 Pyramidal Neurons

**Figure 4:**
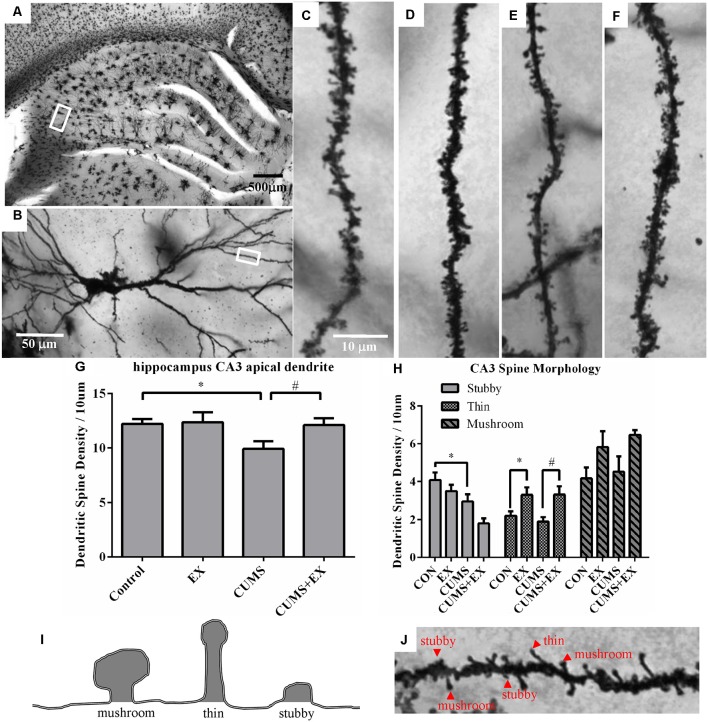
Dendritic spines in the hippocampus CA3 pyramidal neurons were visualized by Golgi staining. Panel **(A)** represents image of the hippocampus. **(B)** Representative microphotograph of a CA3 pyramidal neuron. Representative microphotographs from the marked dendrite showed dendritic spines in the apical dendrites of hippocampal CA3 pyramidal neurons in control **(C)**, EX **(D)**, CUMS **(E)** and CUMS+EX **(F)**. Bar graphs showed density of total dendritic spines **(G)** and spine phenotypes **(H)** in the four groups. Diagram **(I)** and a representative image from the apical dendrites of hippocampal CA3 pyramidal neurons **(J)** showed mushroom, thin and stubby spines. Data represent mean ± SEM. *P < 0.05 CUMS vs. Control or CUMS+EX in **(G)**, *P < 0.05 CUMS vs. control or CUMS+EX in **(H)**, ^#^*P* < 0.05 CUMS vs. CUMS+EX in **(H)**. EX: treadmill exercise. CUMS: chronic unpredictable mild stress (*n* = 5–6 rats/group).

Spine density in the apical dendrites of the hippocampal CA3 pyramidal neurons was 9.9 ± 0.7, 12.1 ± 0.6, 12.4 ± 0.9 and 12.2 ± 0.4 per 10 μm in the CUMS, CUMS+EX, EX and control groups, respectively (Figure 4). To determine the effects of CUMS/exercise and interactions between CUMS and exercise on dendritic spines, we performed two-way ANOVA. Three weeks after CUMS and exercise, CUMS and exercise had a significant effect on spine density (*F*_(3,18)_ = 3.34, *P* = 0.04; Figure 4). In order to analyze whether the interaction between exercise and CUMS has a significant effect on dendritic spines, we fixed one level of a factor and analyzed if the remaining factor is significant. Our analysis showed that without exercise, CUMS cause a significant decrease in spine density compared with a control group (*P* = 0.024); with exercise, CUMS did not cause a significant decrease in spine density compared with a control group. Similarly, without CUMS, exercise alone did not alter spine density (*P* = 0.88); with CUMS, exercise caused a significant increase in spine density compared with CUMS group (*P* = 0.03). These results indicated that treadmill exercise reversed the CUMS-induced decrease in spine density in the apical dendrites of hippocampal CA3 pyramidal neurons (Figure 4G). To determine whether CUMS causes an alteration in spine morphology, we analyzed spine phenotypes including mushroom, thin and stubby spines (Figures 4H–J). Our results are shown below.

#### Stubby Spines

CUMS and exercise had a significant effect on stubby spine density (*F*_(3,17)_ = 6.33, *p* = 0.004). Without exercise, CUMS caused a significant decrease in stubby spine density compared with a control group (*P* = 0.028); with exercise, CUMS also had a significant effect on stubby spine density compared with a control group (*P* = 0.008). However, without CUMS, exercise alone did not affect spine density compared with a control group (*P* = 0.24); with CUMS, exercise did not have a significant effect on stubby spine density compared with CUMS group (*P* = 0.07).

#### Thin Spines

CUMS and exercise had a significant effect on thin spine density (*F*_(3,17)_ = 5.88, *p* = 0.006). No matter with or without exercise, CUMS did not have a significant effect on thin spine density compared with a control group (*P* > 0.05). However, no matter with (*P* = 0.03) or without CUMS (*P* = 0.003), exercise resulted in a significant increase in thin spine density.

#### Mushroom Spines

CUMS and exercise did not have a significant effect on mushroom spines (*F*_(3,17)_ = 2.45, *p* = 0.10). No matter with or without exercise, CUMS did not significantly alter thin spine density (*P* > 0.05). Similarly, no matter with or without CUMS, exercise did not have a significant effect on mushroom spine density (*P* > 0.05).

### Spine Density in the Pyramidal Neurons of the mPFC

**Figure 5:**
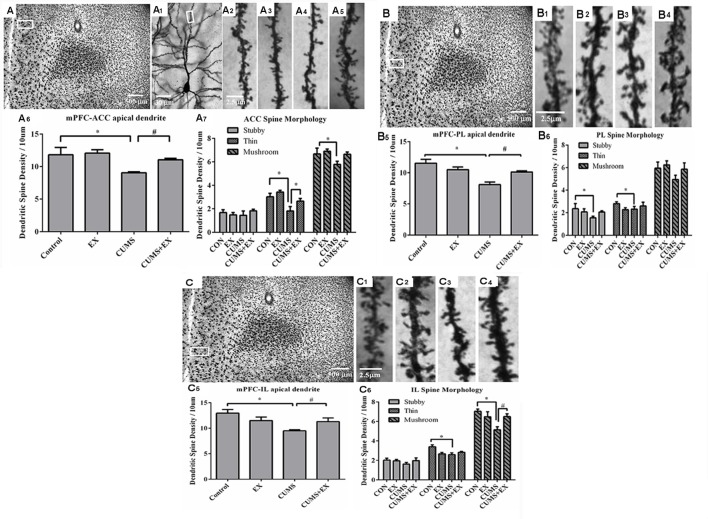
Dendritic spines of the pyramidal neurons in the anterior cingulate cortex (ACC, **A**), prelimbic cortex (PL, **B**) and infralimbic cortex (IL, **C**) of the medial prefrontal cortex (mPFC) were visualized by Golgi staining. Representative microphotographs showed a pyramidal neuron from the boxed ACC **(A_1_)** area of the mPFC. Representative microphotographs showed dendritic spines in the apical dendrites of ACC **(A_2_–A_5_)**, PL **(B_1_–B_4_)** and IL **(C_1_–C_4_)** pyramidal neurons in control **(A_2_,B_1_–C_1_)**, EX only **(A_3_,B_2_–C_2_)**, CUMS only **(A_4_,B_3_–C_3_)** and CUMS+EX **(A_5_,B_4_–C_4_)**, respectively. Bar graphs showed spine density in the pyramidal neurons of the ACC **(A_6,7_)**, PL **(B_5,6_)** and IL **(C_5,6_)**, respectively. Data represent mean ± SEM. *P < 0.05 CUMS vs. Control, ^#^*P* < 0.05 CUMS vs. CUMS+EX. EX, treadmill exercise; CUMS, chronic unpredictable mild stress (*n* = 5–6 rats/group).

Spine density in the apical dendrite of pyramidal neurons in the ACC (Figure 5A), PL (Figure 5B) and IL (Figure 5C) regions of the mPFC was analyzed.

#### ACC Area

Spine density was 9.1 ± 0.2, 11.1 ± 0.2, 12.1 ± 0.5 and 11.8 ± 1.5 per 10 μm in the CUMS, CUMS+EX, EX and control groups, respectively (Figure 5A_1–5_). CUMS and exercise had a significant effect on spine density (*F*_(3,18)_ = 5.76, *P* = 0.006; Figure 5A_6_). Without exercise, CUMS caused a significant decrease in spine density compared with a control group (*P* = 0.04); with exercise, CUMS did not have a significant effect on spine density (*P* = 0.23). Similarly, without CUMS, exercise alone did not affect spine density (*P* = 0.76); with CUMS, exercise caused an increase in spine density compared with CUMS group (*P* = 0.03). These results indicated that treadmill exercise reversed CUMS-induced spine loss in the ACC region of the mPFC.

#### Stubby Spines

CUMS and exercise did not have a significant effect on stubby spine density (*F*_(3,18)_ = 0.42, *p* = 0.74). No matter with or without exercise, CUMS did not have a significant effect on stubby spine density (*P* > 0.05). Similarly, no matter with or without CUMS, exercise did not affect stubby spine density (*P* > 0.05; Figure 5A_7_).

#### Thin Spines

CUMS and exercise had a significant effect on thin spine density (*F*_(3,18)_ = 6.87, *p* = 0.003). Without exercise, CUMS resulted in a significant decrease in thin spine density compared with a control group (*P* = 0.006); with exercise, CUMS did not have a significant effect on thin spine density (*P* = 0.06). Similarly, without CUMS, exercise did not alter thin spine density (*P* = 0.32); with CUMS, exercise caused a significant increase in thin spine density compared with CUMS group (*P* = 0.04). These results showed that exercise reversed CUMS-induced loss in thin spines in the ACC region of the mPFC (Figure 5A_7_).

#### Mushroom Spines

CUMS and exercise did not have a significant effect on mushroom spine density (*F*_(3,17)_ = 3.02, *p* = 0.057). Without exercise, CUMS significantly decreased mushroom spine density (*P* = 0.047); With exercise, CUMS did not alter mushroom spine density (*P* = 0.55). However, no matter with CUMS (*P* = 0.59) or without CUMS (*P* = 0.05), exercise did not have a significant effect on mushroom spine density. These results showed that exercise tended to reverse CUMS-induced loss in mushroom spines, but did not reach statistical significance (Figure 5A_7_).

#### PL Area

Spine density was 8.1 ± 0.4, 10.1 ± 0.2, 10.5 ± 0.5 and 11.5 ± 0.6 per 10 μm in the CUMS, CUMS+EX, EX and control groups, respectively (Figure 5B_1–4_). CUMS and exercise had a significant effect on spine density (*F*_(3,18)_ = 5.76, *P* = 0.0004; Figure 5B_6_). Without exercise, CUMS caused a significant decrease in spine density compared with a control group (*P* < 0.0001); with exercise, CUMS did not have a significant effect on spine density (*P* = 0.56). Similarly, without CUMS, exercise alone did not affect spine density (*P* = 0.13); with CUMS, exercise caused a significant increase in spine density compared with CUMS group (*P* = 0.006). These results indicated that treadmill exercise reversed CUMS-induced spine loss in the PL region of the mPFC (Figure 5B_5_).

#### Stubby Spines

CUMS and exercise did not have a significant effect on stubby spines (*F*_(3,18)_ = 1.59, *P* = 0.23). Without exercise, CUMS resulted in a significant decrease in stubby spine density compared with a control group (*P* = 0.048); with exercise, CUMS did not have a significant effect on stubby spine density (*P* = 0.97). However, no matter with or without CUMS, exercise did not affect stubby spine density. These results showed that exercise tended to reverse CUMS-induced loss in stubby spines, but did not reach statistical significance (Figure 5B_6_).

#### Thin Spines

CUMS and exercise did not have a significant effect on thin spines (*F*_(3,18)_ = 1.59, *P* = 0.34). No matter with or without exercise, CUMS did not have a significant effect on thin spine density. Similarly, no matter with or without CUMS, exercise did not significantly affect mushroom spine density (Figure 5B_6_).

#### Mushroom Spines

CUMS and exercise did not have a significant effect on mushroom spines (*F*_(3,18)_ = 1.62, *P* = 0.22). No matter with or without exercise, CUMS did not have a significant effect on mushroom spine density. Similarly, no matter with or without CUMS, exercise did not significantly affect mushroom spine density (Figure 5B_6_).

#### IL Area

Spine density was 9.5 ± 0.2, 11.3 ± 0.7, 11.5 ± 0.7 and 13.0 ± 0.7 per 10 μm in the CUMS, CUMS+EX, EX and control groups respectively (Figure 5C_1–4_). CUMS and exercise had a significant effect on spine density (*F*_(3,18)_ = 5.76, *P* = 0.004; Figure 5C_5_). Without exercise, CUMS caused a significant decrease in spine density compared with a control group (*P* = 0.0005); with exercise, CUMS did not alter spine density (*P* = 0.82). Similarly, without CUMS, exercise alone did not affect spine density (*P* = 0.11); with CUMS, exercise caused a significant increase in spine density compared with CUMS group (*P* = 0.032). These results indicated that treadmill exercise reversed the CUMS-induced decrease in spine density (Figure 5C_5_).

#### Stubby Spines

Exercise and CUMS did not have the significant effect on stubby spines (*F*_(3,18)_ = 0.96, *P* = 0.43). Exercise, CUMS and the interaction term EX*CUMS are not a significant factor, respectively, and they did not affect stubby spines.

#### Thin Spines

Exercise and CUMS had a significant effect on thin spines (*F*_(3,18)_ = 4.66, *P* = 0.02). Without exercise, CUMS caused a significant decrease in thin spine density compared with a control group (*P* = 0.003); with exercise, CUMS did not alter thin spine density compared with a control group (*P* = 0.51). However, without CUMS, exercise had a significant effect on thin spine density (*P* = 0.006); with CUMS, exercise did not significantly increase thin spine density compared with CUMS group (*P* = 0.32). These results showed that CUMS caused a decrease in thin spine density, which was not reversed by exercise (Figure 5C_6_).

#### Mushroom Spines

Exercise and CUMS had a significant effect on mushroom spines (*F*_(3,18)_ = 4.8, *P* = 0.013). Without exercise, CUMS caused a significant decrease in mushroom spine density compared with a control group (*P* = 0.002); with exercise, CUMS did not cause a significant decrease in mushroom spine density compared with a control group. Similarly, without CUMS, exercise did not have a significant effect on mushroom spine density (*P* = 0.29); with CUMS, exercise resulted in a significant increase in mushroom spine density compared with CUMS group (*P* = 0.02). These results showed that exercise reversed CUMS-induced decrease in mushroom spine density in the IL region (Figure 5C_6_).

### Dendritic Spine Density in the MSNs of the NAc

**Figure 6:**
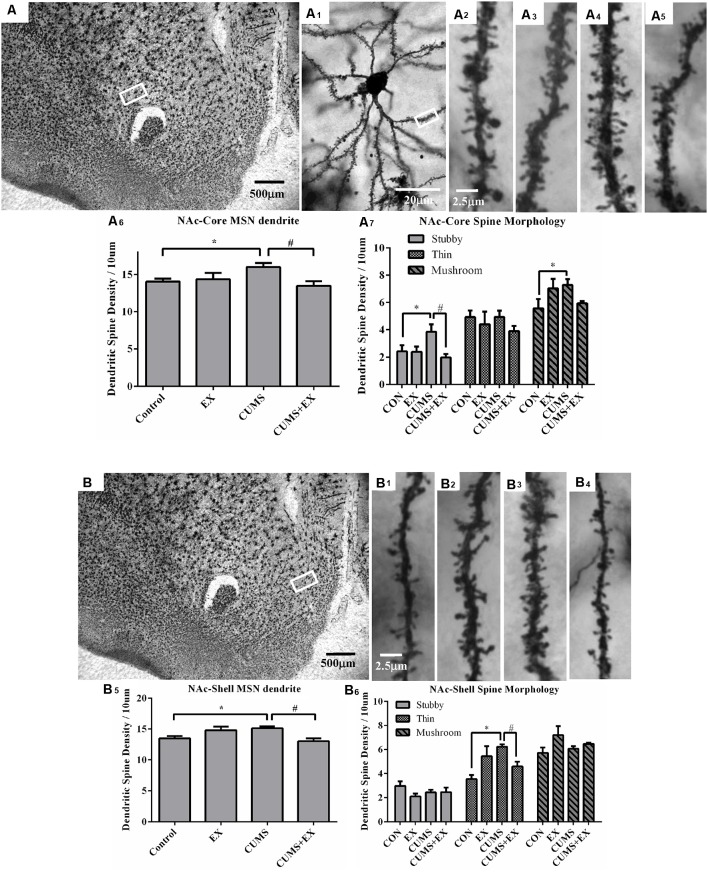
Dendritic spines in the medium spiny neurons (MSNs) of the nucleus accumbens core (NAc-Core) and shell (NAc-Shell) were visualized by Golgi staining. Representative microphotographs showed the NAc core **(A)** and shell **(B)**. A representative microphotograph showed a MSN in the NAc-core **(A_1_)**. Representative microphotographs showed dendritic spines in the MSNs of NAc-Core **(A_2_–A_5_)** and NAc-shell **(B_1_–B_4_)** in control **(A_2_,B_1_)**, EX **(A_3_,B_2_)**, CUMS **(A_4_,B_3_)** and CUMS+EX **(A_5_,B_4_)**. Bar graphs showed spine density in the NAc core **(A_6_,A_7_)** and shell MSNs **(B_5_,B_6_)**. Data represent mean ± SEM. *P < 0.05 CUMS vs. Control, ^#^*P* < 0.05 CUMS vs. CUMS+EX. EX, treadmill exercise; CUMS, chronic unpredictable mild stress (*n* = 5–6 rats/group).

Spine density in the MSNs of the NAc core and shell is presented in Figure 6.

#### NAc Core

The spine density in the MSNs of the NAc core was 16.0 ± 0.5, 13.5 ± 0.6, 14.3 ± 0.9 and 14.0 ± 0.4 per 10 μm in the CUMS, CUMS+EX, EX and control groups, respectively. CUMS and exercise had a significant effect on spine density (*F*_(3,18)_ = 4.08, *P* = 0.024; Figure 6A_6_). Without exercise, CUMS resulted in a significant increase in spine density compared with a control group (*P* = 0.021); with exercise, CUMS did not significantly increase spine density compared with a control group (*P* = 0.27). Similarly, without CUMS, exercise alone did not affect spine density (*P* = 0.71); with CUMS, exercise caused a significant decrease in spine density compared with CUMS group (*P* = 0.004). These results indicated that treadmill exercise reversed CUMS-induced increase in spine density in the MSNs of the NAc core.

#### Stubby Spines

CUMS and exercise had a significant effect on stubby spines (*F*_(3,18)_ = 4.15, *P* = 0.02). Without exercise, CUMS caused a significant increase in stubby spine density compared with a control group (*P* = 0.025); with exercise, CUMS did not cause a significant increase in stubby spine density compared with a control group (*P* = 0.50). Similarly, without CUMS, exercise did not affect stubby spine density (*P* = 0.93), with CUMS, exercise caused a significant decrease in stubby spine density compared with CUMS group (*P* = 0.004). These results indicated that treadmill exercise reversed CUMS-induced increase in stubby spine density (Figure 6A_7_).

#### Thin Spines

CUMS and exercise did not have a significant effect on stubby spines (*F*_(3,18)_= 0.73, *P* = 0.55). No matter with (*P* = 0.99) or without exercise (*P* = 0.54), CUMS did not have a significant effect on thin spine density. Similarly, no matter with (*P* = 0.52) or without CUMS (*P* = 0.21), exercise did not significantly affect thin spine density.

#### Mushroom Spines

CUMS and exercise did not have a significant effect on stubby spines (*F*_(3,18)_ = 2.67, *P* = 0.08). Without exercise, CUMS caused a significant increase in mushroom spine density compared to a control group (*P* = 0.03); with exercise, CUMS did not cause a significant increase in mushroom spine density (*P* = 0.15). However, no matter with CUMS (*P* = 0.06) or without CUMS (*P* = 0.07), exercise did not have a significant effect on mushroom spine density. These results showed that exercise intended to reverse CUMS-induced increase in mushroom spines but did not reach statistical significance (Figure 6A_7_).

#### NAc Shell

The spine density in the MSNs of NAc shell was 15.1 ± 0.3, 13.0 ± 0.5, 14.8 ± 0.6 and 13.5 ± 0.4 per 10 μm in CUMS, CUMS+EX, EX and control groups, respectively (Figure 6B). CUMS and exercise had a significant effect on spine density (*F*_(3,18)_ = 5.28, *P* = 0.009; Figure 6B). Without exercise, CUMS resulted in a significant increase in spine density (*P* = 0.017); with exercise, CUMS did not significantly increase spine density compared with a control group (*P* = 0.051). Similarly, without CUMS, exercise alone did not affect spine density (*P* = 0.055); with CUMS, exercise caused a significant decrease in spine density compared with CUMS group (*P* = 0.003). Similar to the NAc core, treadmill exercise reversed CUMS-induced increase in spine density in the MSNs of NAc shell.

#### Stubby Spines

CUMS and exercise did not have a significant effect on stubby spines (*F*_(3,18)_ = 1.58, *P* = 0.23). No matter with or without exercise, CUMS did not significantly affect stubby spine density. Similarly, no matter with or without CUMS, exercise did not have a significant effect on stubby spine density (Figure 6B_6_).

#### Thin Spines

CUMS and exercise had a significant effect on thin spines (*F*_(3,18)_ = 6.38, *P* = 0.004). Without exercise, CUMS caused a significant increase in thin spine density (*P* = 0.0006) compared with a control group; with exercise, CUMS did not cause a significant increase in spine density compared with a control group (*P* = 0.20). Similarly, without CUMS, exercise had a significant effect on spine density (*P* = 0.009); with CUMS, exercise resulted in a significant decrease in spine density compared with CUMS group (*P* = 0.02). These results showed that treadmill exercise reversed CUMS-induced increase in thin spine density (Figure 6B_6_).

#### Mushroom Spines

CUMS and exercise did not have a significant effect on mushroom spines (*F*_(3,18)_ = 2.42, *P* = 0.10). No matter with or without exercise, CUMS did not have a significant effect on mushroom spine density. Without CUMS, exercise had a significant effect on mushroom spine density (*P* = 0.03), with CUMS, exercise did not significantly alter mushroom spine density (*P* = 0.36).

### Spine Density in the Neurons of Basolateral Amygdala (BLA)

**Figure 7:**
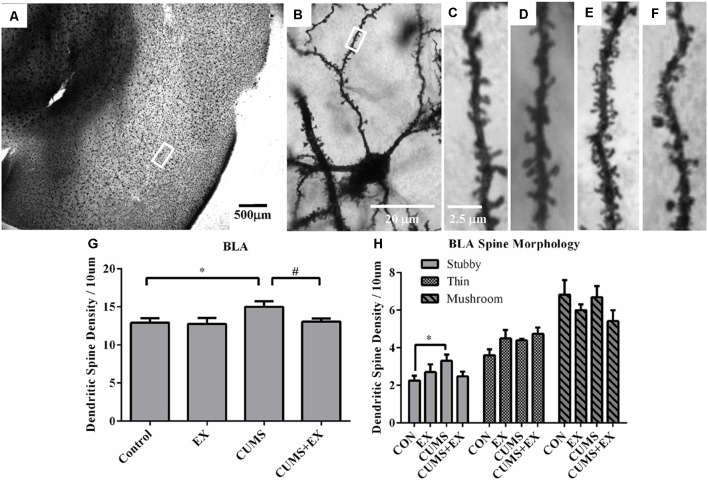
Dendritic spines in the neurons of the basolateral amygdala (BLA) were visualized by Golgi staining. **(A)** A representative microphotograph showed amygdala and **(B)** a representative microphotograph showed a neuron in the BLA. Representative microphotographs showed the changes of dendritic spines in the BLA neurons in control **(C)**, EX **(D)**, CUMS **(E)** and CUMS+EX **(F)** groups. Bar graphs **(G,H)** show spine density in the BLA neurons. The data were expressed as mean ± SEM. *P < 0.05 CUMS vs. Control or CUMS+EX, ^#^*P* < 0.05 CUMS vs. CUMS+EX. EX, treadmill exercise; CUMS, chronic unpredictable mild stress. *n* = 5–6 rats/group.

Spine density in the BLA neurons was 15.0 ± 0.7, 13.1 ± 0.4, 12.8 ± 0.8 and 12.9 ± 0.6 per 10 μm in CUMS, CUMS+EX, EX and control groups, respectively (Figure 7). CUMS and exercise had a significant effect on spine density (*F*_(3,18)_ = 3.11, *P* = 0.049). However, without exercise, CUMS caused a significant increase in spine density compared with a control group (*P* = 0.028); with exercise, CUMS did not significantly increase spine density compared with a control group (*P* = 0.75). Similarly, without CUMS, exercise alone did not affect spine density (*P* = 0.87); with CUMS, exercise caused a significant increase in spine density (*P* = 0.04). These results showed that exercise reversed the CUMS-induced increase in spine density.

#### Stubby Spines

CUMS and exercise did not have a significant effect on stubby spines (*F*_(3,18)_ = 2.26, *P* = 0.12). Without exercise, CUMS caused a significant increase in stubby spine density compared with a control group (*P* = 0.026); with exercise, CUMS did not cause a significant increase in stubby spine density (*P* = 0.61). However, no matter with CUMS (*P* = 0.32) or without CUMS (*p* = 0.68), exercise did not have a significant effect on stubby spine density. These results showed that exercise tended to reverse the CUMS-induced increase in stubby spine density, but did not reach statistical significance (Figure 7H).

#### Thin Spines

CUMS and exercise did not have a significant effect on thin spines (*F*_(3,18)_ = 2.66, *P* = 0.08). No matter with or without exercise, CUMS did not have a significant effect on thin spine density. Similarly, no matter with or without CUMS, exercise did not have a significant effect on thin spine density (Figure 7H).

#### Mushroom Spines

CUMS and exercise did not have a significant effect on mushroom spines (*F*_(3,18)_ = 2.42, *P* = 0.099). No matter with or without exercise, CUMS did not have a significant effect on mushroom spine density. Similarly, no matter with or without CUMS, exercise did not have a significant effect on mushroom spine density (Figure 7H).

## Discussion

Previous studies show that physical exercise is effective in both alleviating depression in human beings (Belvederi Murri et al., 2019) and reversing chronic stress-induced depression-like behaviors in rodents (Chen et al., 2017; Liu et al., 2018). Chronic stress-induced depression-like behaviors in rodents are accompanied by changes in spine density and morphology in the brain (Sousa et al., 2000; Mitra et al., 2005; Radley et al., 2006; Christoffel et al., 2011). In this study, our results showed that CUMS-induced depression-like behaviors were alleviated by exercise and that CUMS-induced alterations in dendritic spines in the principal neurons of the hippocampal CA3 area, mPFC, NAc and BLA were reversed to different extents by exercise. Different spine types may serve different functions, and the alterations in the ratio of these spine types induced by detrimental CUMS- or beneficial exercise may have a different and significant effect on neuronal excitability and associated animal behaviors.

### The Effects of Exercise on CUMS-Induced Depression-Like Behaviors and Body Weight Gain

The core symptom of depression-like behavior, a decrease in sucrose preference, was found in the CUMS-exposed rats compared with the non-stressed controls, which was reversed by treadmill exercise in our study. This is in agreement with previous reports (Wen et al., 2014; Chen et al., 2017). The OFT was used to measure general locomotor activity and exploratory behavior (rearing) in rodents. The CUMS-induced decrease in rearing times in the OFT was reversed by exercise. At the same time, a significant difference in moving distance was not observed among the four groups. Both CUMS and exercise resulted in a decrease in body weight in the CUMS group and exercise group, respectively, compared with the control group which received no stress treatments or exercise, but the CUMS-induced decrease in body weight was greater than the decrease induced by exercise. The CUMS-mediated decrease in body weight in our study is in agreement with reports (Lee et al., 2013; Wen et al., 2014; Chen et al., 2017). However, the exercise-mediated further decrease in body weight in the CUMS-exposed rats in our study does not agree with the report showing that exercise reverses chronic unpredictable stress (CUS)-mediated loss in body weight in the CUS + exercise group compared with the CUS group (Wen et al., 2014). However, some studies report that exercise does not affect the CUS-mediated decrease in body weight in the CUS + exercise group compared with the CUS group without exercise (Lee et al., 2013; Chen et al., 2017). The CUS protocol is similar to CUMS (Willner, 2017). The small discrepancies may be due to the differences in animal species, age, stress and exercise intensity.

### Exercise Reversed CUMS-Induced Spine Loss in the Hippocampus CA3 Area and the mPFC

In the hippocampus, CA3 pyramidal neurons are more sensitive to stress than CA1 neurons (Sousa et al., 2000). Dendritic spines in the apical dendrites of pyramidal neurons in both the hippocampal CA3 and the three regions of the mPFC including the ACC, PL and IL are more sensitive to stress than the spines in the basal dendritic spines (Sousa et al., 2000; Song et al., 2015). Therefore, we analyzed spine density and spine phenotypes in the apical dendrites of these neurons. Although stress-induced dendritic spine loss in these areas has been repeatedly reported (Sousa et al., 2000; Radley et al., 2006; Song et al., 2015), it remains unknown whether exercise could reverse this spine loss and change in spine morphology induced by CUMS. Our study showed that CUMS-induced a decrease in spine density and alterations in spine morphology in the apical dendrites of the pyramidal neurons in the mPFC and hippocampal CA3 area. In comparison with control, treadmill exercise reversed the CUMS-induced decrease in spine density in these brain areas in CUMS + EX groups in comparison with the CUMS group. Furthermore, treadmill exercise did not alter the spine density and morphology in these brain areas in the non-stressed control animals, suggesting that the exercise intensity in this study is appropriate. Importantly, the CUMS-induced decreases in spine density in these brain areas were accompanied by depression-like behaviors, in agreement with previous reports (Li et al., 2011; Qiao et al., 2014). The positive effect of exercise was confirmed by the exercise-mediated increase in thin spine formation in hippocampal CA3 pyramidal neurons in the CUMS + EX group compared with the CUMS group. Exercise-mediated reversal of the CUMS-induced decrease in density of both thin spines in the ACC area and mushroom spines in the IL area may partly contribute to the exercise-mediated alleviation of the depression-like behaviors induced by CUMS. Both thin and mushroom spines are functional spines. To our knowledge, this is the first study showing the beneficial effect of exercise on reversing CUMS-induced alterations in dendritic spines in the hippocampal CA3 area and mPFC.

### Exercise Reversed CUMS-Induced Increase in Spine Density in the NAc and BLA

Dysfunction of the NAc is related to depression (Pizzagalli et al., 2009; Nauczyciel et al., 2013) and depression-like behaviors in rodents (Bessa et al., 2013; Francis and Lobo, 2017). The NAc is divided into the core and shell, which have different functions (Saddoris et al., 2015). CUMS induced an increase in spine density and alterations in spine phenotypes in the MSNs of both the NAc core and shell, in line with the previous report that chronic social defeat stress induces an increase in stubby spine density in the NAc MSNs (Christoffel et al., 2011). Our results also provided evidence for the first time that exercise resulted in a reversal of the CUMS-induced increases in spine density and alterations in spine morphology in the MSNs of both NAc core and shell, which accompanied the alleviation of CUMS-induced depressive-like behaviors. Exercise did not alter the spine density and spine morphology in the non-stressed control animals, but it reversed the CUMS-induced increase in the spine density and changes in spine shape in the CUMS-exposed rats compared with the non-stressed control animals, suggesting a key role of exercise in maintaining normal spine density and morphology in the face of stress.

Abnormally functional connectivity of the amygdala with other brain areas (Cullen et al., 2014; Connolly et al., 2017), elevated activity (Redlich et al., 2017) and morphological abnormalities in the amygdala (Rubinow et al., 2016) are found in patients with depression.

Our present finding of CUMS-induced increases in spine density and alterations in spine shape in the BLA neurons supports the above clinical studies, and is in agreement with a preclinical study showing that chronic immobilization stress induced an increase in spine density in the BLA neurons, which was accompanied by an increase in anxiety-like behavior in rats (Mitra et al., 2005). To our knowledge, this is a novel study that showed a reversal of the CUMS-induced increase in spine density in the BLA neurons by treadmill exercise.

### The Mechanisms of the Reversal of CUMS-Induced Alterations in Spine Density and Spine Morphology by Exercise

The mechanisms through which exercise reversed CUMS-induced alterations in dendritic spines remain unknown. The positive effects of exercise on the hippocampal capillaries, such as the reversal of the stress-induced decrease in the total volume and length of capillaries, as well as the epigenetic regulation of gene expression in the brain, may contribute to the underlying mechanism (Chen et al., 2017; Fernandes et al., 2017). Mitochondria play an essential role in spine formation (Todorova and Blokland, 2017), and protection of the stress-induced dysfunction/over activation of the mitochondria by exercise may play a role in the process (Wen et al., 2014). CUMS-induced depression-like behaviors are accompanied by a decrease in both the ratio of mature BDNF to proBDNF and the level of Kalirin-7 in the hippocampus (Qiao et al., 2017). Mature BDNF increases spine formation and its level is upregulated by exercise, while proBDNF decreases spine formation and its level is down-regulated by exercise (Yang et al., 2014; Park et al., 2017). Exercise-mediated alleviation of CUMS-induced depression-like behaviors, and reversal of CUMS-induced decrease in spine density in the hippocampus, may result partly from reversal of the CUMS-induced decrease in the BDNF level and increase in the pro-BDNF level by exercise (Sigwalt et al., 2011; Park et al., 2017; Shafia et al., 2017). Kalirin-7 is a down-stream target of BDNF (Yan et al., 2016) and plays an essential role in spine formation and controlling spine morphology (Mandela and Ma, 2012). Exercise-mediated reversal of CUMS-induced decrease in the level of kalirin7 protein (Zhuang and Ma et al., unpublished) in the hippocampus may contribute to the underlying mechanism. The underlying mechanisms in the different areas of the brain may be different, which is the focus of our future research.

In summary, CUMS-induced alterations in dendritic spines in the principal neurons of the hippocampal CA3, mPFC, NAc and BLA were accompanied by depression-like behaviors. These alterations were reversed by exercise. Understanding the brain circuit etiology of depression is becoming more and more important, but the underlying mechanisms remain largely unknown. Additional studies are required to determine the molecular mechanisms underlying reversal by exercise of CUMS-induced alterations in dendritic spines in these brain areas of the mood circuit.

## Ethics Statement

This study was carried out in accordance with the recommendations of the ethical principles of animal use and care, the Animal Care and Use Committee of Shaanxi Normal University. The protocol was approved by the Animal Care and Use Committee of Shaanxi Normal University.

## Author Contributions

X-MM designed the experiments. P-CZ, Z-NT and Z-YJ performed the experiments. P-CZ prepared the figures. BW and JG performed the statistical analysis. P-CZ, Z-NT and X-MM wrote the manuscript. All authors approved the final version of the manuscript.

## Conflict of Interest Statement

The authors declare that the research was conducted in the absence of any commercial or financial relationships that could be construed as a potential conflict of interest.

## Footnotes

^1^https://www.who.int/news-room/fact-sheets/detail/depression

## Abbreviations

CUMS: chronic unpredictable mild stress
EX: treadmill exercise
SPT: sucrose preference test
OFT: open field test
ANOVA: analysis of variance
mPFC: medial prefrontal cortex
ACC: anterior cingulate cortex
PL: prelimbic cortex
IL: infralimbic cortex
NAc: nucleus accumbens
MSN: medium spiny neuron
BLA: basolateral amygdala.
